# Efficacy and Safety of Tofacitinib Compared to Cyclophosphamide in Early Diffuse Cutaneous Systemic Sclerosis: A Randomized Controlled Trial

**DOI:** 10.7759/cureus.103163

**Published:** 2026-02-07

**Authors:** Nabil Amin Khan, Ariful Islam, Abu Shahin, Syed Jamil Abdal, Syed Atiqul Haq

**Affiliations:** 1 Department of Rheumatology, Bangladesh Medical University (BMU), Dhaka, BGD; 2 Department of Rheumatology, Bangladesh Medical university (BMU), Dhaka, BGD

**Keywords:** cyclophosphamide, dmards, mrss, systemic sclerosis, tofacitinib

## Abstract

Objective: This study aims to determine the efficacy of tofacitinib in the treatment of early diffuse cutaneous systemic sclerosis.

Materials and methods: This open-label randomized controlled clinical trial was conducted at the Department of Rheumatology, Bangabandhu Sheikh Mujib Medical University, Dhaka. The trial was registered at ClinicalTrials.gov (identifier: NCT06044844). Consecutive sampling was used in this study. Forty-six patients were randomized to groups A and B using block randomization, with 23 patients in each group. In group A, tofacitinib (5 mg) was administered twice daily. Group B received cyclophosphamide (500 mg/m² monthly). Primary efficacy was assessed by the change in the Modified Rodnan Skin Score (mRSS) from baseline after 24 weeks. Secondary efficacy was assessed by the change in the Disease Activity Score for 28 joints (DAS28) in response to changes in C-reactive protein (CRP) level and erythrocyte sedimentation rate (ESR). The Bangla version of the Health Assessment Questionnaire-Disability Index (B-HAQ) response from baseline to 24 weeks was analyzed. Oral prednisolone (≤10 mg/day), calcium channel blockers, and phosphodiesterase 5 inhibitors (sildenafil and tadalafil) were administered. Follow-up was performed at four, 12, and 24 weeks. History, physical examination, and relevant investigations were used to assess adverse effects. Changes in acute-phase reactants and composite measures within the groups from baseline to 24 weeks were also analyzed. Adverse effects were assessed through patient history, physical examination, and relevant investigations.

Results: Per-protocol analysis showed a significantly greater reduction in mRSS in group A compared with group B at 12 weeks (7.0 ± 2.89 vs. 5.26 ± 2.42; p = 0.03) and 24 weeks (10.17 ± 2.92 vs. 8.0 ± 4.08; p = 0.04). DAS28-ESR and DAS28-CRP reductions were significant between the groups from baseline to weeks 12 and 24 (p < 0.05). The reduction in functional status (measured by B-HAQ) was 2.11 ± 4.91 and 0.96 ± 0.53 in groups A and B, respectively (p = 0.43). FVC change was 9.17 ± 8.33 in group A and 3.43 ± 8.1 in group B. Within the other core set of outcomes, composite measures were significantly improved in group A. Two patients (8.7%) in group A and six patients (17.4%) in group B developed nausea. Two patients (8.7%) in both groups developed respiratory tract infections and urinary tract infections. Taeniasis developed in three patients (13%) in group B. Two cyclophosphamide-treated patients (8.7%) developed hemorrhagic cystitis.

Conclusions: Tofacitinib demonstrated significant efficacy in reducing skin thickness (mRSS) in early dcSSc, showing both numerical and statistical advantages over cyclophosphamide at 24 weeks, along with a more favorable safety profile. These findings support further evaluation of tofacitinib as a potential therapeutic option in this patient population.

## Introduction

Systemic sclerosis (SSc) is a multi-system inflammatory disease in which dysregulated fibrosis, autoimmunity, and vasculopathy lead to disability, organ failure, and accelerated mortality. SSc has two main subtypes: limited cutaneous (lc) SSc and diffuse cutaneous (dc) SSc, with the disease subtype determined by the distribution of skin involvement and different patterns of internal organ involvement observed in the two subtypes. SSc has an estimated prevalence of 276 per million in the United States; 10-year mortality rates range from 23% to 45% [1]. SSc has very high morbidity and mortality rates among all rheumatic diseases [2]. Progressive skin fibrosis in systemic sclerosis is a surrogate marker of visceral organ progression and mortality [3]. The Modified Rodnan Skin Score (mRSS) is a measure of skin thickness and has been used as the primary outcome measure in clinical trials of diffuse cutaneous SSc (hereafter referred to as diffuse SSc or dcSSc) [4]. Currently, there are no definitive, effective disease-modifying agents for this potentially devastating condition. Although cyclophosphamide, methotrexate, and mycophenolate mofetil are commonly used, there remains an urgent need for more effective therapies for systemic sclerosis. The available treatment options include methotrexate, mycophenolate mofetil, and cyclophosphamide as monotherapy. Despite the availability of many drugs, cyclophosphamide is effective for the treatment of both skin thickening and interstitial lung disease (ILD). However, none of the patients responded equally to the cyclophosphamide therapy. Recent evidence shows that the Janus kinase (JAK)/signal transducer and activator of transcription (STAT) signaling pathway is markedly activated in patients [5]. In genetic studies, variants in the STAT locus were strongly associated with SSc [6]. Therefore, JAK/STAT signaling may play a crucial role in the pathogenesis of SSc.

Tofacitinib inhibits JAK1/JAK3 and is effective in rheumatoid arthritis, psoriatic arthritis, and ulcerative colitis; it is also a new therapeutic option for systemic lupus [7]. However, the efficacy of JAK inhibitors in patients with SSc remains under investigation. Blocking JAK/STAT activity with tofacitinib abrogates core fibrotic responses in fibroblasts and prevents multiple-organ fibrosis in mice [8]. These findings are the first to demonstrate that in SSc patients with genomic evidence of enhanced JAK/STAT pathway activity in target organs, tofacitinib treatment might be effective in slowing or reversing fibrosis [9]. Increased JAK2 activation has also been detected in the skin of patients with SSc, particularly in fibroblasts [10]. Therefore, we compared the efficacy of tofacitinib with that of cyclophosphamide in treating dcSSc.

This study was previously presented as a poster (abstract no: 1584) on the efficacy of tofacitinib in early diffuse cutaneous systemic sclerosis at ACR Convergence 2024, Poster Session B, on November 17, 2024 [11].

## Materials and methods

Study population

Patients aged at least 18 years with systemic sclerosis were diagnosed according to the 2013 American College of Rheumatology (ACR)/European League Against Rheumatism (EULAR) classification criteria. Patients with a diffuse cutaneous subtype of systemic sclerosis, a disease duration of less than 60 months, and an mRSS between 10 and 45 were included in this study. Patients were excluded from the study if they had a recent or concurrent infection, including active tuberculosis; hemoglobin <9 g/dL; total white blood cell count <4000/µL; neutrophil count <1200/µL; lymphocyte count <750/µL; platelet count <100 x 109/mm3; alanine aminotransferase or aspartate aminotransferase >3 × the upper limit of normal; estimated glomerular filtration rate <60 mL/min/1.73 m2; pulmonary disease with forced vital capacity (FVC) ≤35% of predicted; and a history of malignancy. Pregnant or breastfeeding women of childbearing potential who did not use highly effective contraception were excluded from this study.

Study design

This open-label randomized controlled clinical trial was conducted at the Department of Rheumatology, Bangabandhu Sheikh Mujib Medical University (BSMMU), Dhaka, from February 2023 to May 2024. Skin thickening was considered the primary entry criterion for this study. Patients were enrolled consecutively, and randomization was performed using block randomization. The subjects in both groups were vaccinated in accordance with the ACR guidelines for adults.

Patients were enrolled based on the 2013 ACR/EULAR classification criteria for SSc [12]. The study specifically focused on individuals with early dcSSc, a phase during which aggressive immunomodulatory therapy, such as tofacitinib, may confer the most significant benefit before irreversible fibrosis develops. Previous studies suggest that early-stage disease (typically within three to five years of onset) is more amenable to such targeted treatments [13]. By limiting the study to ≤60 months, the study ensures participants are at similar disease stages, reducing variability in skin thickening and organ involvement.

A latent tuberculosis test was performed at baseline. Patients at least 18 years of age with systemic sclerosis were diagnosed using the ACR/EULAR classification criteria in 2013 [12]. If the test was positive, the patient was treated with a three-month course of rifampicin and isoniazid (per the recommendation for latent tuberculosis treatment) and enrolled in this study after three months. Sample size was calculated using this formula: $n = \frac{(Z_{\alpha/2} + Z_{\beta})^2 \times 2\sigma^2}{(\mu_1 - \mu_2)^2}$. Forty-six patients were randomized to groups A and B using block randomization, with 23 in each group. If any patient was taking methotrexate, three weeks were given to wash out the drug [10]. The subjects in both groups were vaccinated for adult vaccination in accordance with the ACR guidelines [14].

In our study, we used the Bengali version of the Health Assessment Questionnaire (B-HAQ). This version has already been published in a peer-reviewed journal. The specific questionnaire used in our research is also included as an annexure [15].

In group A, tofacitinib (5 mg) was administered twice daily. Group B received cyclophosphamide 500 mg/m² monthly. Oral corticosteroids (≤10 mg/day prednisolone), calcium channel blockers, and phosphodiesterase 5 inhibitors (sildenafil and tadalafil) were used. All patients underwent baseline evaluation before treatment, including the mRSS, B-HAQ, DAS28-ESR, and laboratory tests (CBC, CRP, SGPT, serum creatinine), chest X-ray (posteroanterior view), and the Mantoux test. Follow-up will be performed at 4, 12, and 24 weeks [15]. An mRSS was used to evaluate treatment response. Efficacy was assessed at the end of the 24th week using mRSS [16]. Adverse effects were assessed based on medical history, physical examination, and investigations.

However, the tool assesses general functional domains such as dressing, arising, eating, walking, hygiene, reach, grip, and activities of daily living, which are not disease-specific but instead reflect the overall physical ability of patients with chronic rheumatic diseases. Several studies have validated the HAQ in SSc populations, demonstrating good reliability, construct validity, and sensitivity to change. The HAQ is also frequently used as an outcome measure in clinical trials and observational studies of SSc [17].

The Health Assessment Questionnaire-Disability Index (HAQ-DI) was translated into Bengali using a rigorous forward-backward translation process. The translated version underwent cognitive pretesting with 30 outpatients, followed by field testing with 100 patients, to assess internal consistency and construct validity. To ensure cultural relevance, ten items were adapted to reflect the Bengali context better. The final Bengali version (B-HAQ) demonstrated strong internal consistency and construct validity in psychometric validation. These findings indicate that the B-HAQ is a reliable and valid tool for assessing functional disability among Bengali-speaking patients with rheumatoid arthritis and other rheumatic diseases.

However, the tool assesses general functional domains such as dressing, arising, eating, walking, hygiene, reach, grip, and activities of daily living, which are not disease-specific but instead reflect the overall physical ability of patients with chronic rheumatic diseases. Several studies have validated the HAQ in SSc populations, demonstrating good reliability, construct validity, and sensitivity to change. The HAQ is also frequently used as an outcome measure in clinical trials and observational studies of SSc and is endorsed by groups such as the EULAR Scleroderma Trials and Research Group.

Statistical analysis

Data were processed and analyzed using SPSS Statistics version 27 (IBM Corp. Released 2020. IBM SPSS Statistics for Windows, Version 27.0. Armonk, NY: IBM Corp.). Patient demographics were summarized as counts and percentages for qualitative data, means ± standard deviations (SDs) for normally distributed quantitative data, and medians ± interquartile ranges (IQRs) for skewed distributions. Quantitative variables (baseline and 12- and 24-week observations) between groups were compared using an unpaired t-test and a within-group paired t-test. The Mann-Whitney U test was used to compare non-normally distributed observations between groups, and the Wilcoxon signed-rank test was used within groups. A chi-square test was used to assess the association between the qualitative variables. Statistical significance was set at p ≤ 0.05.

**Figure 1:**
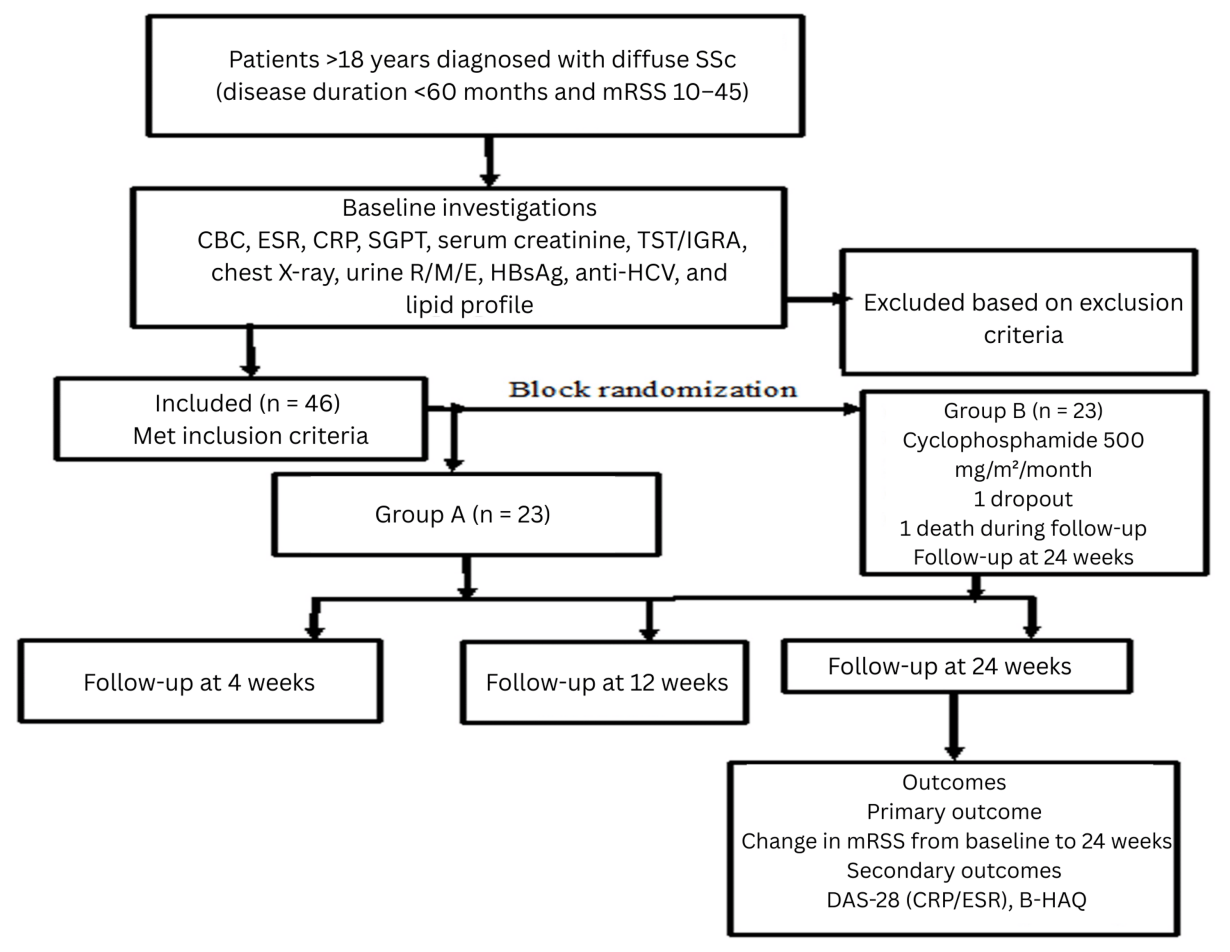
Flowchart showing the baseline information, randomization, and follow-up records of the patients Pt: patient, SSc: systemic sclerosis, mRSS: Modified Rodnan Skin Score, CBC: complete blood count, ESR: erythrocyte sedimentation rate, CRP: C-reactive protein, SGPT: serum glutamic pyruvic transaminase, TST: tuberculin skin test, IGRA: interferon-gamma release assay, R/M/E: routine and microscopic examination, HBsAg: hepatitis B surface antigen, Anti-HCV: antibody to hepatitis C virus, F/U: follow-up, DAS-28: Disease Activity Score for 28 joints, B-HAQ: Bangla version of the Health Assessment Questionnaire-Disability Index

**Figure 2:**
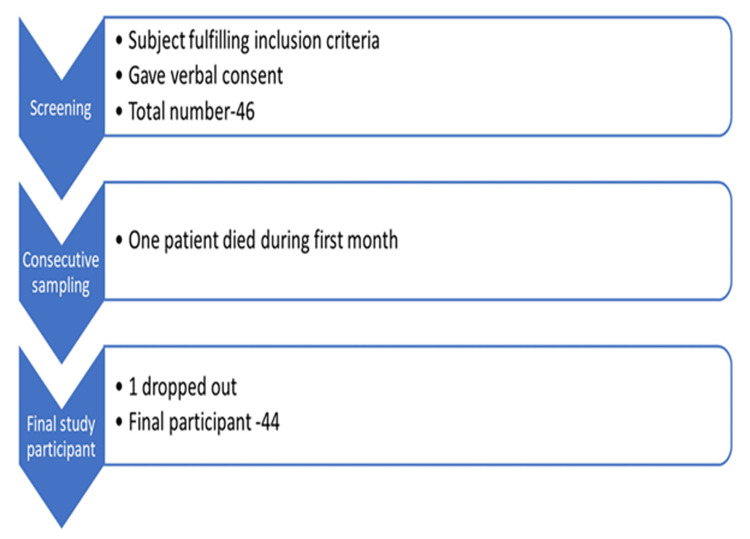
Flowchart showing the screening of the patients

Figure 1 illustrates the study procedure through a flowchart, while Figure 2 depicts the patient screening process and the final number of participants included in the study.

## Results

A total of 46 patients were screened for enrollment in accordance with the inclusion criteria. Twenty-three patients were in group A, and 23 were in group B. One patient in group B was lost to follow-up, and one died. A total of 44 patients completed the 24-week study. Per protocol, analysis was performed at baseline and at the end of the 12th and 24th weeks.

Among the study subjects (n = 46), the mean ages were 32.22 ± 8.35 and 39.86 ± 9.28 years in groups A and B, respectively. In group A, 20 (87%) were female, and three (13%) were male; in group B, 21 (91.3%) were female, and two (8.7%) were male. The mean BMI (kg/m²) in group A was 19.7 ± 4.4, and in group B it was 19.5 ± 2. The mean duration between symptom onset and enrollment for the study was 14.7 ± 6.5 and 15.3 ± 7.87 months in groups A and B, respectively. Most of the patients in both groups were housewives. Mean mRSS were 28.96 ± 5.29 in group A and 26.39 ± 5.96 in group B. In group B, the mean DAS28-CRP was 2.91 ± 1.2, and 2.35 ± 1.11 in group B. The mean DAS28-ESR was 3.6 ± 1.22 and 3.16 ± 1.18 in groups A and B. The mean CDAI was 8.44 ± 9.03 and 5 ± 7.77 in groups A and B. The mean FEV1 was 61.78 ± 19.8 and 67.91 ± 10.63, and the mean FVC was 62.21 ± 19.73 and 71.47 ± 12.14 in groups A and B. There was no statistically significant difference between tender joint count, patient global assessment, and physician global assessment. Functional disability measured by the B-HAQ was higher in group A than in group B (2.38 ± 4.73 vs 1.23 ± 0.43; p = 0.25). There were no significant differences in the baseline ESR and CRP levels between the groups. The baseline sociodemographic characteristics, core set of outcomes, and composite measures are shown in Table 1.

**Table 1:** Baseline sociodemographic characteristics, disease activity (CDAI, DAS28-ESR, CRP), and physical functioning/disability (BHAQ) of study participants (n = 46) Data are expressed as frequency (%) or mean ± SD. A p-value ≤0.05 was considered statistically significant. Baseline includes all 46 participants; one patient in group B died, and one was lost to follow-up (final N = 44). * statistically significant, # lower level of significance, ## higher level of significance BMI: body mass index, SD: standard deviation

Characteristics	Group A (n = 23)	Group B (n = 23)	p-value
	Mean ± SD	Mean ± SD	
Age group (years)	32.22 ± 8.35	39.87 ± 9.28	0.005#
Gender			
Male, n (%)	3 (13.0)	2 (8.7)	0.64*
Female, n (%)	20 (87.0)	21 (91.3)	
BMI (kg/m^2^)	19.7 ± 4.4	19.5 ± 2.0	0.85^# ^
Disease duration (years)	14.7 ± 6.5	15.3 ± 7.87	0.81^# ^
Educational level			
No formal education, n (%)	1 (4.3)	7 (30.4)	0.002*
Below secondary, n (%)	8 (34.8)	13 (56.5)	
Above secondary, n (%)	14 (60.9)	3 (13.0)	
Occupation			
Housewife, n (%)	10 (43.5)	21 (91.3)	0.002*
Service, n (%)	9 (39.1)	2 (8.7)	
Unemployed, n (%)	4 (17.4)	0 (0.0)	
Modified Rodnan Skin Score (mRSS)	28.96 ± 5.29	26.39 ± 5.96	0.13^# ^
Disease Activity Score for 28 joints using C-reactive protein (DAS28-CRP)	2.92 ± 1.22	2.34 ± 1.11	0.11^##^
Disease Activity Score for 28 joints using erythrocyte sedimentation rate (DAS28-ESR)	3.69 ± 1.22	3.16 ± 1.18	0.14^#^
Clinical Disease Activity Index (CDAI)	8.44 ± 9.03	5 ± 7.77	0.19^## ^
Forced vital capacity (FVC)	62.22 ± 19.73	71.48 ± 12.14	0.06^# ^
Tender joint count (TJC)	4.3 ± 4.45	2.52 ± 4.02	0.16^##^
Patient Global Assessment of Disease Activity (PtGA)	2.56 ± 2.52	1.48 ± 2.21	0.13^## ^
Physician Global Assessment of Disease Activity (PhGA)	1.74 ± 1.69	1 ± 1.62	0.14^## ^
Erythrocyte sedimentation rate (ESR)	39 ± 21.09	37.13 ± 18.5	0.75^## ^
C-reactive protein (CRP)	7.75 ± 6.19	6.98 ± 6.11	0.68^## ^
Bangla version of the Health Assessment Questionnaire-Disability Index (B-HAQ)	1.28 ± 0.43	1.44 ± 0.45	0.22^##^

The mean change in the core set of outcomes and composite measures was assessed between groups from baseline to the 12th week and is shown in Table 2. The mean reduction in mRSS was 7 ± 2.89 in group A and 5.26 ± 2.42 in group B. DAS28-CRP and DAS28-ESR were significantly different between the groups. The other significant outcomes were the TJC and PhGA. There were no significant changes in PtGA, ESR, CRP, B-HAQ, and CDAI between the groups (Table 2).

**Table 2:** Assessment of mean changes from baseline in clinical and laboratory variables between groups at week 12 (n = 44) Data are expressed as mean ± SD. Group A included 23 participants (n = 23), and group B included 21 participants (n = 21). A p-value ≤0.05 was considered statistically significant. Baseline analysis included all 46 participants; one patient in group B died, and one was lost to follow-up, resulting in a final sample size of 44. # specific, marked difference, ## stronger, more statistically significant difference SD: standard deviation

Variables	Group A (n = 23) mean ± SD	Group B (n = 21) mean ± SD	p-value
Modified Rodnan Skin Score (mRSS)	7 ± 2.89	5.26 ± 2.41	0.03^# ^
Disease Activity Score for 28 joints using C-reactive protein (DAS28-CRP)	0.72 ± 0.99	0.22 ± 0.69	0.02^##^
Disease Activity Score for 28 joints using erythrocyte sedimentation rate (DAS28-ESR)	0.88 ± 1.03	0.29 ± 0.83	0.01^##^
Clinical Disease Activity Index (CDAI)	5.22 ± 6.34	2.04 ± 4.13	0.05^##^
Tender joint count (TJC)	2.86 ± 3.33	1 ± 2.05	0.03^##^
Patient Global Assessment of Disease Activity (PtGA)	1.61 ± 1.9	0.65 ± 1.4	0.05^## ^
Physician Global Assessment of Disease Activity (PhGA)	1.2 ± 1.24	0.44 ± 0.89	0.03^##^
Erythrocyte sedimentation rate (ESR)	2.91 ± 16.96	1.26 ± 17.26	0.72^##^
C-reactive protein (CRP)	0.79 ± 4.52	0.31 ± 3.14	0.22^## ^
Health Assessment Questionnaire–Disability Index (HAQ-DI)	0.78 ± 0.56	1.96 ± 4.89	0.36^##^

The mean change in the core set of outcomes and composite measures was assessed between the groups from baseline to the 24th week and is shown in Table 3. The mean reduction in mRSS was 10.17 ± 2.92 in group A and 8 ± 4.07877 in group B, with a significant difference (p < 0.05). The DAS28-ESR score was significantly different between the groups. The change in FVC was also significant. There were no significant differences in the other core outcome sets or composite measures between the groups (Table 3).

**Table 3:** Comparison of mean reduction in variables between groups from baseline to week 24 (n = 44) Data are expressed as mean ± SD. Group A included 23 participants (n = 23) and group B included 21 participants (n = 21). A p-value ≤0.05 was considered statistically significant. # specific, marked difference, ## stronger, more statistically significant difference SD: standard deviation

Variables	Group A (n = 23) mean ± SD	Group B (n = 21) mean ± SD	p-value
Modified Rodnan Skin Score (mRSS)	10.17 ± 2.92	8 ± 4.08	0.04^# ^
Disease Activity Score for 28 joints using C-reactive protein (DAS28-CRP)	0.88 ± 0.97	0.42 ± 0.8	0.09^## ^
Disease Activity Score for 28 joints using erythrocyte sedimentation rate (DAS28-ESR)	0.86 ± 0.81	0.45 ± 0.87	0.04^##^
Clinical Disease Activity Index (CDAI)	5.69 ± 6.5	3.3 ± 5.21	0.23^## ^
Forced vital capacity (FVC)	9.17 ± 8.33	3.43 ± 8.1	0.02^##^
Tender joint count (TJC)	3 ± 3.15	1.65 ± 2.69	0.1^## ^
Patient Global Assessment of Disease Activity (PtGA)	1.83 ± 2.1	1 ± 1.62	0.2^##^
Physician Global Assessment of Disease Activity (PhGA)	1.22 ± 1.38	0.65 ± 1.03	0.17^##^
Erythrocyte sedimentation rate (ESR)	2.61 ± 15.91	4.87 ± 20.22	0.72^##^
C-reactive protein (CRP)	0.63 ± 3.59	0.76 ± 5.33	0.22^# ^
Health Assessment Questionnaire–Disability Index (HAQ-DI)	2.11 ± 4.91	0.96 ± 0.53	0.43^##^

Group B had more adverse events than group A over 24 weeks. The most common adverse events in group A were fever (8.7%), nausea (8.7%), respiratory tract infection (RTI; 8.7%), and an increased lipid profile. In group B, the most common adverse events were fever (26.1%), nausea (8.7%), urinary tract infection (UTI; 8.7%), RTI (8.7%), taeniasis (13%), and hemorrhagic cystitis (8.7%) (Table 4).

**Table 4:** Assessment of adverse effects between groups at week 24 (n = 46) Data are expressed as frequency (%). Group A included 23 participants (n = 23) and group B included 23 participants (n = 23). A p-value ≤0.05 was considered statistically significant. * statistically significant

Variables	Group A (n = 23)	Group B (n = 23)	p-value
Fever	2 (8.7)	6 (26.1)	0.12*
Nausea	2 (8.7)	4 (17.4)	0.38*
Urinary tract infection (UTI)	2 (8.7)	2 (8.7)	1*
Respiratory tract infection (RTI)	2 (8.7)	2 (8.7)	1*
Taeniasis	0 (0.0)	3 (13.0)	0.07*
Hemorrhagic cystisis	0 (0.0)	2 (8.7)	0.15*

## Discussion

This study aimed to assess the efficacy and safety of tofacitinib and cyclophosphamide in patients with early diffuse cutaneous systemic sclerosis. Skin involvement in diffuse SSc predicts the extent of visceral involvement, prognosis, and mortality [3]. The mRSS reflects disease progression; improvement in the mRSS is a positive prognostic sign, whereas worsening is associated with a poorer prognosis [18]. Therefore, mRSS was selected as the primary efficacy endpoint. Additionally, we evaluated musculoskeletal involvement because articular and periarticular involvement has been proven to be associated with a more aggressive disease course, progression of skin fibrosis, internal organ involvement, and worse prognosis [18].

There are limited options for treating skin involvement in systemic sclerosis, including methotrexate, mycophenolate mofetil, and cyclophosphamide. The efficacy of methotrexate in treating diffuse and progressive skin diseases has been evaluated in two randomized, placebo-controlled, double-blind trials. Higher doses of methotrexate (25 mg/week) were more effective but were associated with increased side effects. However, there is no evidence that methotrexate is effective for visceral organ involvement.

The cost of mycophenolate mofetil is unaffordably high [19]. According to the European Scleroderma Observational Study recommendations, cyclophosphamide has demonstrated efficacy in improving skin thickening in systemic sclerosis and has been evaluated in multiple clinical trials [20-24] for cutaneous manifestations. Since over 40% of patients with systemic sclerosis have ILD [25], and ILD is particularly common in diffuse cutaneous SSc, cyclophosphamide was selected as the comparator drug in this study. This choice was further supported by its dual benefit for both skin and lung involvement, despite recent guidelines favoring methotrexate, mycophenolate mofetil, or rituximab for skin manifestations.

The European Scleroderma Observational Study [26] showed a significant reduction in mRSS with cyclophosphamide use (mean reduction 5.7). Another study comparing cyclophosphamide pulse therapy with autologous stem cell transplantation reported an 8.8-point reduction in mRSS. According to post hoc analysis of scleroderma lung studies 1 and 2, mRSS reduction with cyclophosphamide was 5.4 and 7.2 after 12 and 24 months, respectively. Cyclophosphamide is also effective against lung and cardiac involvement in systemic sclerosis. The percentage of side effects in oral versus intravenous cyclophosphamide was 44% versus 23%, respectively. A weighted regression model showed 12- and 24-month survival of 91.7% and 90.1%, respectively, in cyclophosphamide-treated patients [27].

In this study, significant between-group differences favoring tofacitinib were also observed in DAS28-ESR at both time points and in DAS28-CRP at 12 weeks. Differences in other composite measures, including CDAI, were not statistically significant. No statistical difference in the B-HAQ response was observed between groups A and B at baseline. Group A had a higher baseline mRSS than group B. The composite measures measured by DAS28-ESR, DAS28-CRP, and CDAI were higher in group B. In this study, the primary core outcome, mRSS, showed a statistically significant improvement in group A compared with group B at both 12 and 24 weeks. In our study, we found that both drugs were effective within the group in improving disease signs and symptoms, disease activity (measured by composite indices), and physical function. We found that the improvement in skin thickness by mRSS from baseline to the 12th week was 7 ± 2.89 in group A and 5.26 ± 2.42 in group B. At the end of the 24th week, the difference from baseline was 10.17 ± 2.92 and 8 ± 4.1 in group A and group B, respectively. By 24 weeks, both groups achieved clinically meaningful responses, defined as ≥25% improvement in mRSS [22].

A similar study compared tofacitinib with methotrexate in diffuse cutaneous SSc [8]. The results were similar to those of our study. Our results indicate that the mean changes in FEV1 and FVC were 7.57 and 9.17 in group A and 3.91 and 3.44 in group B. Both of these changes were significant. In another study, the mean change in FVC from baseline was 2.88 and 2.19 in cyclophosphamide- and mycophenolate-treated patients, respectively [28]. Our study had some limitations. The route of administration of cyclophosphamide, the smaller sample size, and the shorter period could all have contributed to the difference relative to that study. The mean reduction of DAS28-CRP and DAS28-ESR from baseline to the 24th week was 0.87 ± 0.97 and 0.42 ± 0.81 between group A and group B, which is insignificant. However, within the group, the mean reductions in DAS28-CRP and DAS28-ESR from baseline to the 24th week were significant. Functional improvement (assessed by B-HAQ ≥0.22 improvement) was greater in group A than in group B, which was statistically significant. There was no significant difference in the ESR and CRP levels between the groups. There was also no significant change in PASP or EF% between or within the groups from baseline to 24 weeks.

Regarding safety, our findings add to essential pharmacovigilance data for chronic immunosuppressive therapies. Although tofacitinib demonstrated a more favorable adverse event profile, cyclophosphamide was associated with higher rates of infection and hemorrhagic cystitis. These observations reinforce the need for vigilant monitoring for rare but serious drug-associated complications during long-term treatment of chronic systemic diseases, documented in the case of gastrointestinal perforation among rheumatoid arthritis patients receiving tofacitinib, tocilizumab, or other biologic treatment. The most common adverse events in group A were fever (8.7%), nausea (8.7%), RTI (8.7%), and raised lipid profile. In group B, the most common adverse events were fever (26.1%), nausea (8.7%), UTI (8.7%), RTI (8.7%), taeniasis (13%), and hemorrhagic cystitis (8.7%). One patient in group B died during the first month of treatment. The patient had no signs of infection or cardiac symptoms. This was probably a natural death, as the event occurred at home during sleep. I could not obtain a proper history or examination.

This single-center study requires validation in a larger multicenter cohort. It was unblinded, had a small sample size, and had a short duration. Although this trial was initially planned in early 2022, participant enrollment began only after obtaining IRB approval on 29 January 2023 and subsequent registration at ClinicalTrials.gov. The study period from November 2023 to May 2024 resulted in a relatively short recruitment window, which may have limited the sample size. Moreover, it lacked sufficient power to detect differences in individual adverse events. The primary outcome measure was mRSS, but it has limitations, including inter- and intra-rater variability.

## Conclusions

In this 24-week comparative study, both tofacitinib and cyclophosphamide were effective in reducing disease activity and improving clinical outcomes, with significant improvements observed in mRSS, DAS28-ESR, and FVC. Tofacitinib demonstrated a statistically significant greater reduction in the primary endpoint, mRSS, compared with cyclophosphamide, along with significant improvements in DAS28-ESR and FVC. While differences in other measures, such as CDAI, were not significant, the overall efficacy and more favorable safety profile suggest that tofacitinib is a promising therapeutic alternative for early dcSSc. Both treatments were generally well tolerated; however, adverse events were more frequent in the cyclophosphamide group, including fever, gastrointestinal symptoms, urinary tract infections, and taeniasis. Overall, tofacitinib may provide a favorable efficacy and safety profile compared to cyclophosphamide in this patient population.
